# Can user testing of a clinical trial patient information sheet make it fit-for-purpose? - a randomized controlled trial

**DOI:** 10.1186/1741-7015-9-89

**Published:** 2011-07-21

**Authors:** Peter Knapp, David K Raynor, Jonathan Silcock, Brian Parkinson

**Affiliations:** 1 Department of Health Sciences, University of York, YO10 5DD, UK; 2 School of Healthcare, University of Leeds, Leeds, LS2 9LN, UK; 3 School of Pharmacy, University of Bradford, Bradford, BD7 1DP, UK; 4 Making Sense Design, 15 Paternoster Row, Sheffield, S1 2BX, UK

## Abstract

**Background:**

The participant information sheet (PIS) provided to potential trial participants is a critical part of the process of valid consent. However, there is long-standing concern that these lengthy and complex documents are not fit-for-purpose. This has been supported recently through the application of a performance-based approach to testing and improving readability called user testing. This method is now widely used to improve patient medicine leaflets - determining whether people can find and understand key facts. This study applied for the first time a controlled design to determine whether a PIS developed through user testing had improved readability over the original, using a sheet from a UK trial in acute myeloid leukemia (AML16).

**Methods:**

In the first phase the performance of the original PIS was tested on people in the target group for the trial. There were three rounds of testing including 50 people in total - with the information revised according to its performance after each of the first 2 rounds. In the second phase, the revised PIS was compared with the original in a parallel groups randomised controlled trial (RCT) A total of 123 participants were recruited and randomly allocated to read one version of the PIS to find and show understanding of 21 key facts.

**Results:**

The first, developmental phase produced a revised PIS significantly altered in its wording and layout. In the second, trial phase 66% of participants who read the revised PIS were able to show understanding of all aspects of the trial, compared with 15% of those reading the original version (Odds Ratio 11.2; Chi-square = 31.5 *p *< .001). When asked to state a preference, 87.1% participants chose the revised PIS (Sign test *p *< .001).

**Conclusions:**

The original PIS for the AML16 trial may not have enabled valid consent. Combining performance-based user testing with expertise in writing for patients and information design led to a significantly improved and preferred information sheet. User testing is an efficient method for indicating strengths and weaknesses in trial information, and Research Ethics Committees and Institutional Review Boards should consider requesting such testing, to ensure that PIS are fit-for-purpose.

## Background

There have been two areas of recent activity with regards to clinical trials that might be seen to be in opposition. On the one hand in some countries there has been a governmental drive to increase trial activity, in both the number of trials being conducted and the proportion of patients taking part [1]. On the other hand there have been increasing concerns expressed about the conduct of trials, in relation to participant safety (particularly in Phase 1 trials) and the process and quality of informed consent. These concerns were increased and articulated strongly following the 2006 TGN1412 trial incident at Northwick Park, London, in which six healthy volunteers became seriously ill [2] and in the high-level reports that followed [3-6]. The reports were consistently critical of the inadequacies of information giving and the consent process in trials.

In many countries participant consent to clinical research, including trials, is based on two forms of information provision: a written Patient Information Sheet (the PIS); and spoken information, usually from a clinician. Research has documented the patchy quality of both of these aspects. For example, studies looking at patients' understanding at the end of a trial have found sub-optimal comprehension, such as one in five participants not knowing the name of the medicine being tested [7] and similar proportions not knowing that they could withdraw at any time [8,9]. These findings are confirmed by a systematic review of communication and informed consent in cancer trials [10] in which aspects such as treatment risks and benefits and the right to withdraw consent were found to be not well understood. The review concluded that 'patients do not appear to be adequately informed' (p.304). In two observational studies of patients being recruited to trials key aspects were missing from spoken information provided by clinicians and participants' understanding of the trial (which was sometimes erroneous) was not checked or corrected [11,12].

A lack of participant knowledge might result from the difficulty in understanding complex information, such as randomization [13], or because the PIS is poorly written or too complex. The level of literacy required to read the PIS is often too high for the general population [14,15]. Poor information provision may particularly affect older patients and those with fewer years of education [16].

Research into the quality of PIS has used either a checklist method - looking for the presence of features that might enhance or inhibit reading - or readability formulae, such as the Simple Measure of Gobbledygook (SMOG) or Flesch-Kincaid [17-19] - as indicators of readability. These methods mostly concentrate on word and sentence length and disregard layout, which may be an influence on readability [20], and they offer a hypothetical rather than an actual measure of readability. At best they provide only a partial indication of a document's readability and, given the important elements of a document that they do not measure, they might be seen to mislead.

Ancker and colleagues [21] argue that a PIS should be analyzed according to its performance, rather than on a number obtained from a readability formula. We recently adopted a method of performance-based testing - user testing - to assess the ability of three PIS to inform people from the target group for the trial [22-24]. The study findings were consistent: the existing sheets (which had been approved by NHS research ethics committees) were flawed in their ability to allow people to find and understand important aspects of the trial in relation to safety, efficacy, consent and practical details. In each of the three studies the PIS had both its layout and wording significantly revised and further user testing revealed improvements in its performance.

However, weaknesses of this previous research were twofold: the studies included small sample sizes, and the study design (that is, serial testing and refinement) allowed the possibility that (although the cohorts were matched by age and education) better performance of the PIS was due to sample characteristics and not improvements in the sheet itself. The study reported here aimed to overcome these methodological weaknesses to give an estimate of the size of effect of improving the readability of a trial PIS, in this case the PIS used in a recent trial of treatments in AML16. The study comprised two phases: (1) a developmental phase and (2) a trial phase.

## Developmental phase: method

### Design

An independent groups design was used, with each participant seeing only one version of the information.

### Participants

Fifty members of the public were recruited using local publicity (flyers and newspaper adverts) to take part in readability studies. Participants were adults aged 55 and over, to mimic recruitment to the actual AML16 trial. Exclusion criteria were: having taken part in any medicine trial or readability study in the previous six months; close personal experience of leukemia; healthcare professionals. We ensured that each round of ten participants had a similar profile in terms of two likely influences on testing, age and education.

### Tested materials

**Figure 1:**
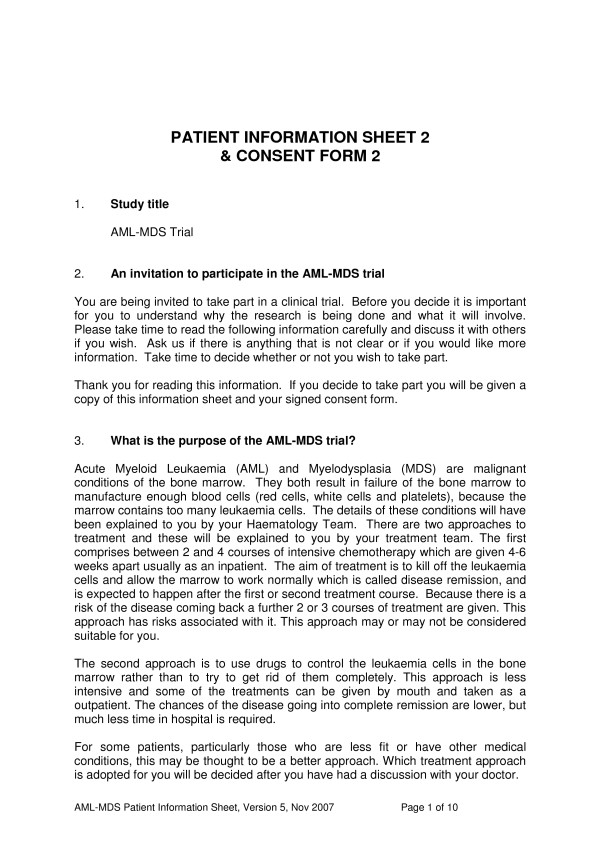
**Example page of the original AML16 Participant Information Sheet**.

The first PIS tested was the original AML16 trial PIS, comprising ten pages of single-sided A4 paper and containing 3,200 words of text (see Figure 1 for an example section) was obtained from the AML trials website (http://www.aml16.bham.ac.uk). We contacted the authors to inform them of the proposed study, and asked for confirmation about how it was presented to potential participants. Before testing, all content identifying individuals or organizations was replaced with pseudonyms.

The second tested material was a first revision of the AML16 trial sheet, retaining its meaning but with revised layout, appearance, structure and wording - informed by the results from the first round of testing.

In the third round of testing a second revision of the trial sheet, informed by the results from the second round of testing, was evaluated.

### Outcomes

Participants' ability to find 21 key points of information in the sheets, and then show their understanding of each of those points (see Table 1). The 21 items were drawn from four categories that apply to trials of any phase: the nature and purpose of the trial (4 questions); the process and meaning of consent (4 questions); trial procedures (6 questions); and safety and efficacy of the tested medicine (7 questions).

**Table 1:** Information finding and understanding results from the trial phase

	Original version (n = 55)		Revised version (n = 61)	
	Found	If found, understood	Found	If found, understood
**Nature and purpose of the trial**				
**Q4 **What sort of patient is being offered 'non-intensive' treatment?	45	45	58	58
**Q14 **What is this trial trying to find out?	24	18	54	49
**Q15 **How will it be decided which patients in the trial receive which treatment?	52	52	61	61
**Q20 **A patient could get 1 of 4 different treatments within the trial. What are the 4 different treatments?	55	52	60	59
**Process and meaning of consent**				
**Q2 **Suppose you decided to take part in the trial but later changed your mind. Would you have to give a reason?	55	55	59	59
**Q8 **Suppose you take part and are harmed by the trial. What can you do?	54	54	60	60
**Q16 **What happens to the information collected about you as part of the trial?	55	54	61	60
**Q17 **Suppose you decide not to take part in the trial and receive the standard treatment. What medicines would you be given?	37	36	58	58
**Trial procedures**				
**Q1 **If you need to contact the Haematology Unit at the hospital, what number should you use?	49	47	61	61
**Q3 **Suppose you receive the drug Trisenox in the trial. How and when would it be given to you?	55	55	61	61
**Q7 **What does the sheet say about the blood tests you would need to have if you took part in the trial?	35	34	58	57
**Q10 **During the trial you may need to receive transfusions. Of what?	55	54	61	61
**Q12 **When you receive any of the treatments in this trial, how far apart are the courses of treatment?	54	53	61	61
**Q21 **If you don't take part in the trial and receive low dose Cytarabine as the 'standard treatment'. How is it given to you and how often?	53	49	61	60
**Safety and efficacy of the medicine**				
**Q5 **What should you do to try to stop yourself getting a cold or infection while in this trial?	53	52	60	60
**Q6 **Which parts of the body are most likely to be affected by side effects of the medicines in this trial?	54	54	61	60
**Q9 **The medicine Mylotarg can cause jaundice. What does the information say about jaundice and what you should do?	54	54	60	60
**Q11 **What does the sheet say about contraception while taking part in this trial?	55	55	58	58
**Q13 **What does the information say about driving to and from the hospital for treatment?	55	55	60	60
**Q18 **After each course of treatment the doctor may want to examine your bone marrow. Why?	45	41	60	59
**Q19 **Imagine you are taking part in the trial and you get an infection. What should you do?	52	51	60	60
**Total items NOT found**	**109/1155**		**28/1281**	
**Total items found but NOT understood**		**26**		**11**

The authors independently selected the key points for questions, based on the pre-defined categories, with any differences reconciled by consensus, and the questionnaire was then written. Questions were arranged so that their order did not correspond with the order of the information in the sheet. During testing, each of the 21 items was scored for finding information (yes or no) and, if found, scored for understanding (yes or no).

Participants' evaluative comments on the participant information sheets were recorded in the second part of the interview, as was the time taken to read the information sheet and the time taken for the structured user testing questions.

## Procedure

This phase comprised 3 stages:

1. Testing of the original information sheet. The information was tested using participants who were instructed to imagine receiving treatment for leukemia and being asked to take part in a trial to test different drug treatments. They were posted the sheet so that they received it at least 24 hours before testing and were asked to read it carefully (and note how many minutes it took them to read it). When they attended for testing they were given a chance to read it once more (with the reading time recorded) and were left alone to do so. Then each of the 21 user test questions was put in turn and the participant was asked, first, to find the answer in the sheet and, second, to give their answer and, where required, to explain what the information meant. The interviewer judged if the participant had demonstrated understanding by their answer to each question; any uncertainties in scoring were resolved through later discussion among the interviewers and PK, by using the interview transcript. No upper time limit was placed on answering each question and the interviewer moved on to the next question when the participant provided an answer, or if the participant gave up and requested to move on, or when it became clear that they could not find the answer. After the 21 structured questions participants were asked for their general impressions of the sheet, with particular focus on the appearance, wording, print size, headings and organization of information. Interviews were audio recorded and transcribed.

2. Re-wording and re-design of the Information Sheet. Revision of the information was based on three sources: participants' user test questionnaire data and their opinions; good practice in information wording and design [24]; the authors' experience and expertise in information writing and design. Care was taken to retain the original meaning of the information, and no information was removed, except in the case of repetition.

3. Testing of the revised Information Sheet (as per stage (1), followed again by participants' evaluative comments.

### Data analysis

Data analysis looked at: the scores for each of the 21 questions to determine how many participants could find and then understand the answer to each question and could at least eight of ten participants do so, to match European Commission legislative thresholds for licensed medicine leaflets [25]; how many participants could answer all of the 21 questions correctly (that is, score a clear round); and participants' evaluative comments after the formal questioning.

## Results

Testing of the original PIS was conducted on 20 participants during March 2009, following 4 pilot interviews, using the 21 item user test questionnaire as reported in Table 2. Among the 21 questions, 3 questions (7, 17, 21) had significant problems, with fewer than 16 of 20 participants being able to find the answer and show understanding. Only 3 of the 20 participants (15%) were able to find and show understanding for all 21 questions (clear round). Problems identified were that some aspects of the trial were not explained clearly (for example, participants struggled to understand whether there were three, four or five arms to the trial) or the sheet used technical language that participants found difficult. In addition, the PIS was densely printed and text heavy. The layout was hard to follow; for example, sub-sections were not well indicated and the hierarchy of headings was unclear.

**Table 2:** Participant characteristics in the trial phase

	**Original PIS**^**a**^	Revised PIS	Original PIS	Revised PIS
**Sex**	19 m/43 f	23 m/38 f	18 m/37 f	23 m/38 f
**Age**	Mean 63.9 years (IQR^b ^58-65)	Mean 65.7 years (IQR 62-68)	Mean 64.1 years (IQR 58-66)	Mean 65.7 years (IQR 62-68)
**Highest educational attainment (1 = completed by 16;** **2 = by 18 or equivalent;** **3 = graduate)**	1 = 31 2 = 18 3 = 3	1 = 31 2 = 13 3 = 17	1 = 26 2 = 17 3 = 12	1 = 31 2 = 13 3 = 17
	n = 62	n = 61	n = 55	n = 61

Randomized (n = 123); interviewed (n = 116)

^a^PIS, patient information sheet;^b^IQR, Inter-quartile range,

Testing of the first revised version was conducted on 10 participants during April 2009, following 4 pilot interviews. Of the 21 questions, 1 question scored fewer than 8 out of 10 participants finding and showing understanding. Four of the ten participants scored a clear round (40%). Participants were experiencing some problems with understanding the allocation of treatments and finding information about data storage. As a result, testing was halted after 10 participants and changes were made to the sub-headings. For example, the sub-heading 'Will my taking part in this trial be kept confidential?' was changed to 'What will happen to information about me collected during the trial?'

Testing of the second revised version was conducted on 20 participants in April and May 2009. Participants were better able to find and understand information in the PIS; interview times were reduced and participants' evaluations of the PIS were more positive. On only 1 of the 21 questions did fewer than 16 out of 20 participants find and show understanding of the information. Ten of the 20 participants scored a clear round (50%).

We now had a revised version of the PIS which user testing suggested had improved readability. The second phase compared the original sheet with the improved sheet in a controlled study to determine whether this confirmed the difference and to estimate the size of effect.

## Trial phase: method

### Design

A controlled trial design was used, featuring parallel groups, two arms, with stratified, random allocation by individual.

### Participants

A total of 123 members of the public were recruited using local publicity (flyers and newspaper adverts) to take part in readability studies. As for the developmental phase, participants were adults at least 55 years old chosen to mimic recruitment to the actual AML16 trial, and the exclusion criteria were also the same.

### Allocation

Participants were allocated to one of two groups (original or revised PIS) by random number sequence with permuted blocks of four. Allocation was stratified according to two factors that may impact on the ability to find and understand information during testing: age (55 to 69; 70+); and education (completing education at 16 or under; completing at 18 or equivalent; being a higher education graduate). After consent was taken, the recruiting researcher phoned a remote center to receive the allocation.

### Tested materials

**Figure 2:**
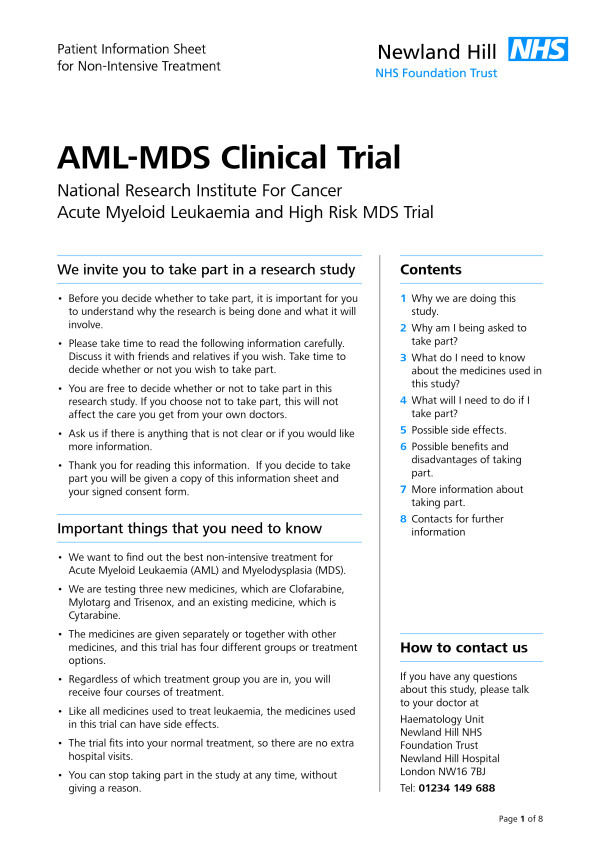
**Page 1 of the revised AML16 Participant Information Sheet**.

**Figure 3:**
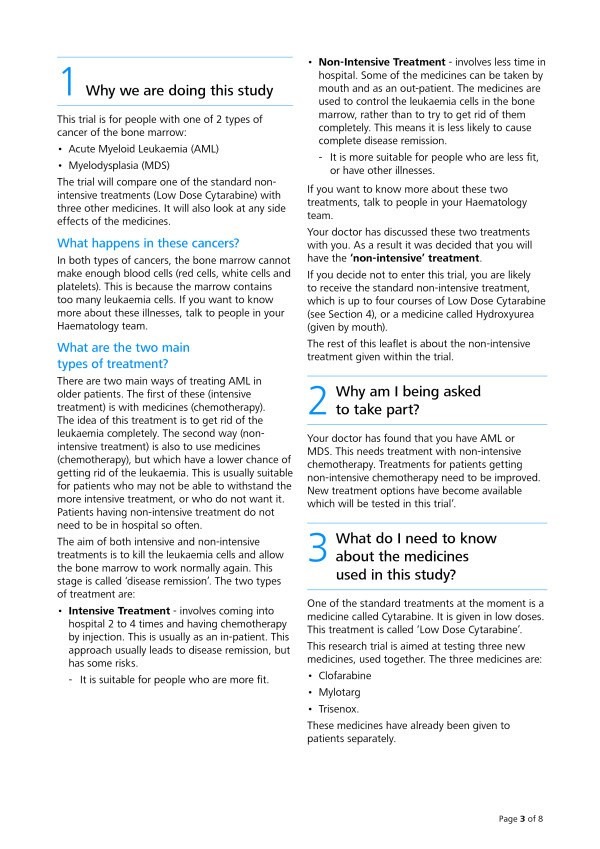
**Page 3 of the revised AML16 Participant Information Sheet**.

Two PIS were tested: (1) the original AML16 trial participant information sheet, as described above (see Figure 1); and (2) a revised version of the AML16 trial sheet, developed and tested as described above, retaining its meaning but with revised format, appearance and wording (for examples see Figures 2 and 3).

The revised version was printed double-sided on two sheets of A3 paper, then folded to form a birthday card style booklet of A4 size. Text was printed using Linotype Frutiger Next black font and blue headings were used to contrast with the body text. Page numbers were inserted, as were clear sub-headings (denoted by use of larger, bold text). Bullets and numbers were used for lists. A contents list was placed on the front page, as well as a headline section summarizing key points in the sheet. The main text of the sheet was divided into eight sections (Why we are doing this study; Why am I being asked to take part?; What do I need to know about the medicines used in this study?; What will I need to do if I take part?; Possible side effects; Possible benefits and disadvantages of taking part; More information about taking part; Contacts for further information).

Changes to the wording included shortening sentences and paragraphs, as well as replacing difficult or technical words with a lay alternative. Examples include: from 'disease going into complete remission' to 'getting rid of the leukemia'; from 'nausea and vomiting' to 'feeling and being sick'; and from '...doctor will check your blood chemistry' to '...doctor will do blood tests'.

When technical terms were required in order to be precise, we first used a lay alternative and followed it with the original term in parentheses. Examples include: 'given slowly into a vein (intra-venous infusion)'; and 'injection under the skin (sub-cutaneous)'.

We also gave greater prominence to the contact names and telephone numbers and placed them on both the first and last pages.

### Outcomes

As for the developmental phase, we were interested in participants' ability to find 21 key points of information in the sheets, and then convey their understanding of each of those points (see Table 1). The primary outcome measure was the proportion of participants who could find and understand all 21 aspects.

Participants' preferences for one of the two participant information sheets were recorded, as was the time taken to read the information sheet and the time taken to complete all the structured user testing questions.

### Procedure

The procedure followed that used for the developmental phase, but with two additions. First, after the recruitment phone call to a participant, the researcher phoned a telephone randomization center to receive the allocation. The PIS was then posted, ensuring that the participant would receive it at least 24 hours before testing.

Second, at the end of testing, participants were shown the PIS version that they had not been tested on, and were asked to read it briefly. They were asked to state which of the two versions they preferred and why.

### Sample size and data analysis

We based the sample size of the trial phase on the proportion of participants who would be able to find and understand answers to all 21 questions. Based on data from the developmental phase, we estimated that the revised PIS would result in twice as many participants having a clear round (estimated proportions were 25% for the original and 50% for the revised versions) - a difference we considered meaningful in terms of valid consent. With 90% power a sample size of 116 participants would be needed at the *p *< .01 level of probability.

The variables of interest were: the number of participants having a clear round (that is, finding and understanding all 21 answers); the mean number of items either not found or not understood; the mean total reading time; the mean time taken for the 21 structured user testing questions; and participants' preferences for the PIS version.

### Research ethics

Approval to conduct both phases of the study was granted by the University of Leeds, School of Healthcare Research Ethics Committee in May 2009.

## Trial phase: results

### Participants

**Figure 4:**
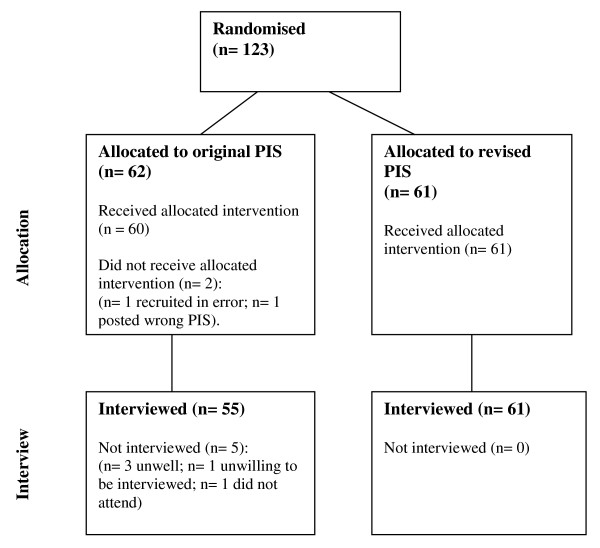
**Trial flow diagram**.

A total of 123 people were randomized, of whom 116 were interviewed. Two were withdrawn due to protocol violation (one was under 55 years of age; the other was posted the wrong PIS version) and five did not attend (three were unwell on the day; one found the PIS upsetting and did not want to be interviewed; one did not attend and could not be contacted). All seven participants not interviewed, had been randomised to the original PIS group. Hence the analyses are based on 116 interviewed participants, as guided by the sample size calculation (55 in the original group and 61 in the revised group), see Figure 4.

Following three pilot interviews, 116 participants were interviewed during June to August 2009.

### Finding and understanding all 21 answers (clear round)

Eight participants (14.5%) in the original PIS group were able to find and understand all 21 answers, whereas 40 participants (65.6%) in the revised PIS group were able to do so. The difference is statistically significant.

Original 8/55

Revised 40/61

Chi-square = 31.5; *P *< .001; Odds Ratio = 11.2

### Finding and understanding scores

Participants' ability to find answers in the PIS to the questions (see Table 1) was associated with the version they had read. On average those reading the original version could not find 1.9 items (standard error (SE) 0.2; range 0 to 6); in the revised version group the mean was 0.4 items (SE 0.2; range 0 to 10). The difference (mean 1.5; 95% Confidence Intervals (CIs) 1.0 to 2.0) is statistically significant (F = 4.73; *p *= 0.032).

Participants understood almost all of the information they had found. Their ability to understand answers to questions was not associated with the sheet they had read. In the original version group they could not understand 0.3 items (SE 0.1; range 0 to 3), whereas in the revised version group they could not understand 0.2 items (SE 0.1; range 0 to 3). The difference (0.1; 95% CIs -0.1 to 0.4) is not statistically significant (F = 1.92; *p *= 0.17).

### Reading and question times

Participants' reading times were calculated by adding their self-recorded home reading time and their reading time before testing (when applicable). All participants reported reading the sheet at home and had recorded their reading time, which ranged from 10 to 90 minutes. A total of 48 participants (41.4%) chose to read the sheet again immediately before testing (and this additional reading time ranged from 2 to 25 minutes). Total reading time did not vary according to group (original version 27.6 minutes, range 14 to 90, SE 1.5; revised version 28.4 minutes, range 11 to 79, SE 1.7; F = .030, *p *= .86).

The time taken to complete the 21 structured user test questions varied greatly among the participants and also varied according to allocation. Participants reading the original PIS needed 42.4 minutes (range 16.0 to 72.3; SE 1.7) whereas those reading the revised PIS needed 26.4 minutes (range 13.4 to 58.2; SE 1.3). The mean difference of 16.1 minutes (95% CIs 12.0 to 20.2) was statistically significant (F = 60.1; *p *< .001).

### Sheet preference

Participants' stated preference also showed a significant difference between the two versions. Fifteen participants (12.9%) preferred the original version, whereas 101 participants (87.1%) preferred the revised (Sign test *p *< .001).

## Discussion

User testing of the AML16 participant information sheet showed that it performed sub-optimally. Participants could not find or took a long time to find some of the information, but when information was found, it was almost always understood. Only a small minority of participants were able to find and show understanding of all aspects of the AML16 trial from the original version of the PIS. The revised version performed much better. Time taken in interviews was much shorter, illustrating the difficulty faced in finding answers by those in the original version group, and almost two-thirds of participants were able to find and show understanding of all aspects of the AML16 trial. When asked to compare the two sheets and state a preference, almost all participants preferred the revised version.

The sheet had been significantly revised during the developmental phase, by noting its performance during user-testing, and re-writing and re-designing to address the problems identified, using good practice in information design and clear writing. The contribution of user-testing as a developmental tool appears crucial - for example, in indicating continued weaknesses and in confirming when changes to the sheet have had a meaningful effect. These weaknesses could not have been identified solely through expert review.

The results confirm the pattern reported in three smaller, uncontrolled studies [22-24]. This study confirms that it is possible to improve written trial information (both in terms of its ability to inform and its appeal) by a combination of testing by lay people, along with the application of clear writing and information design. There was no difference between the two versions in the amount of information understood, but readers first have to locate information in order to understand it. This finding may indicate the greater contribution made by revision of the structure and layout of the PIS, as compared to changes in wording. However, to determine conclusively the relative effects of wording revisions and design revisions would require a different study design to that used here.

In several aspects of the study there were significant differences between what took place and what would happen to patients actually being asked to consider trial participation. The participants were members of the public asked to imagine themselves in a situation, participants were able to read the PIS at home before testing, and finally the user testing interviews took place in a quiet interview room rather than a busy hospital or clinic. While these factors may have aided participants in their reading, it is unlikely to have biased the data in favor of either version. Also in contrast to an actual trial, participants were asked to show an understanding of the AML16 trial from the PIS alone, without access to spoken information from a recruiting clinician. It is unclear whether the presence of a clinician would narrow differences between the two versions of the PIS, or whether clinicians might in fact provide clearer spoken information when being able to refer to a PIS that was more clearly written and designed. This would be worthy of further research.

As mentioned in the introduction Ancker [21] and others argue that a PIS should be analyzed according to how it performs, rather than by a number obtained from a readability formula, a view supported by this study. US Institutional Review Boards often require a certain formula score to be obtained before a trial sheet is approved. Formulae can indicate the difficulty of language within a document, but they offer very limited data in terms of which aspects of a document work and which do not. The user testing scores obtained for the original AML16 PIS would question whether a participant in the trial would have been able to give valid or informed consent, particularly since only one in seven people could find and understand answers to all questions.

The study examined participants' ability to find and understand written information, rather than test their willingness to participate in a trial. This study shows convincingly that a participant dependent only on written information would be more able to use it effectively, when it has been written and designed with its purpose in mind, as argued by Jefford and Moore [26]. However the effect on actual trial participants is an important question. If using improved sheets resulted in increased trial recruitment rates, this would be both meaningful and valuable. Thus the effectiveness (and cost-effectiveness) of improving participant information materials is a question that requires an answer. A recent systematic review of interventions to increase trial recruitment reported that different forms of information had no impact [27]. What is not clear from the review is whether enhanced information failed to impact on knowledge, or whether it did increase knowledge but failed to change behavior - an important distinction that deserves clarification.

## Conclusions

Combining the use of user testing by lay people with expertise in writing for patients and information design resulted in a greater proportion of participants being able to find and understand information about the trial. Not only would this impact on the extent to which valid consent is given, but it may also impact on the recruitment rate and patient behavior within the trial. Research ethics committees and Institutional Review Boards should consider requesting user testing to provide assurance that PIS are meeting the needs of people being recruited to clinical trials.

## List of abbreviations

AML: acute myeloid leukaemia; CIs: confidence intervals; NHS: National Health Service; PIS: participant information sheet; RCT: randomised controlled trial; SE: standard error

## Competing interests

D K Raynor is a director of Luto Research Ltd, a University of Leeds spin-out company that provides information writing and testing services to the pharmaceutical industry, and which recruited participants to the study. Brian Parkinson provides graphic design services to the pharmaceutical industry and the NHS.

## Authors' contributions

PK, DKR and JS conceived the study idea, designed the method and wrote the revised participant information sheet. BP designed the revised information sheet. PK managed the data collection and analysis. All authors contributed to writing the article.

## Pre-publication history

The pre-publication history for this paper can be accessed here:

http://www.biomedcentral.com/1741-7015/9/89/prepub
